# Biomarkers of diabetes risk in the National Diet and Nutrition Survey rolling programme (2008–2011)

**DOI:** 10.1136/jech-2013-202885

**Published:** 2013-09-19

**Authors:** S Almoosawi, D Cole, S Nicholson, I Bayes, B Teucher, B Bates, J Mindell, S Tipping, C Deverill, A M Stephen

**Affiliations:** 1 MRC Human Nutrition Research, Elsie Widdowson Laboratory, Cambridge, UK; 2 German Cancer Research Center, Heidelberg, Germany; 3 National Centre for Social Research, London, UK; 4 University College London, London, UK

**Keywords:** DIABETES, EPIDEMIOLOGY, DIET, NUTRITION

## Abstract

This study describes the distribution of glycosylated haemoglobin (Hb_A1c_) and glucose concentrations in the combined year 1 (2008–2009), year 2 (2009–2010) and year 3 (2010–2011) of the National Diet and Nutrition Survey (NDNS) rolling programme. The NDNS rolling programme is a nationally representative survey of food consumption, nutrient intakes and nutritional status of people aged 1.5 years and over living in England, Wales, Scotland and Northern Ireland. The study population comprised survey members who completed three or four days of dietary recording and who provided a blood sample. After excluding survey members with self-reported diabetes (n=25), there were 1016 results for Hb_A1c_ and 942 for glucose (not the same individuals in each case). Around 5.4% of men and 1.7% of women aged 19–64 years, and 5.1% of men and 5.9% of women aged ≥65 years had impaired fasting glucose (glucose concentrations 6.1–6.9 mmol/L). Over 20% of men aged ≥65 years had fasting glucose concentrations above the clinical cut-off for diabetes (≥7 mmol/L) compared to 2.1% of women of similar age (p=0.007). Similarly, 16.4% of men had Hb_A1c_ concentrations ≥6.5%, compared to 1.5% of women (p=0.003). Children and teenagers had fasting glucose and Hb_A1c_ values largely within the normal range. To conclude, this is the first study to provide data on the distribution of Hb_A1c_ and glucose concentrations in a nationally representative sample of the British population. The high prevalence of men aged ≥65 years with Hb_A1c_ and glucose concentrations above the clinical cut-off of diabetes warrants further attention.

## Introduction

The incidence of type 2 diabetes has risen dramatically around the world in recent decades and is set to increase further. It has been estimated that the number of people with diabetes worldwide will increase from 171 million in 2000 to over 2600 million in 2030.^1^ In England, the number of people diagnosed with diabetes has risen with an estimated 2.4 million people reported to have diabetes in 2010–2011^2^; increases in prevalence have been noted in recent successive years: 5.1% in 2008–2009, 5.3% in 2009–2010 and 5.5% in 2010–2011.^3^ This is in contrast to the prevalence of cardiovascular disease (CVD) and stroke, both of which show a relatively steady prevalence for all age groups in the UK, as assessed by the new Quality and Outcomes Framework of the National Health Service (NHS).^4^ Diabetes affects particular subgroups of the population. It is more common in the elderly,^5–7^ certain ethnic groups such as South Asians,^7–9^ whose risk is much greater in the UK than in their country of origin, and the obese. Currently in the UK, diabetes and associated complications costs the NHS £4.9 billion/year, about one-tenth of its total budget. Complications of diabetes include a number of serious conditions such as nephropathy, neuropathy, renal failure, myocardial infarction, blindness, leg ulcers and amputations.^10^ ^11^

The National Diet and Nutrition Survey (NDNS) programme is a nationally representative survey of children and adults from age 1.5 years and above living in private households in the UK. In 2002/2003, following a review, the Food Standards Agency decided to move to a rolling programme, where all age groups from 1.5 years to the elderly would be assessed in an ongoing and rolling programme of surveillance. Although markers of diabetes risk have traditionally not been included in NDNS, the rising prevalence of the disease, as well as the complications and disease entities associated with it, warranted their inclusion in the NDNS rolling programme. Markers of diabetes risk have been included in the National Health and Nutrition Examination Surveys (NHANES) in the USA since the 1980s, and are now considered a fundamental measure in this programme.^12^ In England, glycated haemoglobin (Hb_A1c_) has been measured on occasion in the Health Survey for England (HSE).^13^ However, this survey does not provide data for all the countries of the UK. The NDNS rolling programme provides a unique opportunity to examine glucose status because of its national representatives where markers of glucose status are collected in a representative UK sample covering different socioeconomic groups and all ages from 1.5 years and upwards. Moreover, since NDNS is planned to run on a rolling basis, it will be possible to study temporal trends in Hb_A1c_ and fasting glucose. Finally, NDNS collects detailed dietary information using a 4-day estimated diary, making it one of the few surveys in the UK where it will be possible to examine relationships between diet including nutrients, foods and dietary patterns in relation to Hb_A1c_ and fasting glucose.

The aim of this study was to present the findings for the blood markers of diabetes risk from the first 3 years of the NDNS rolling programme (2008–2011), and to describe the prevalence of diagnosed and undiagnosed diabetes.

## Methods

### Study population

The NDNS rolling programme is a nationally representative survey of food consumption, nutrient intakes and nutritional status of people aged 1.5 years and over living in England, Wales, Scotland and Northern Ireland.^14^ The survey was commissioned by the UK Food Standards Agency in 2006 and carried out by a consortium of three organisations: the National Centre for Social Research (Nat Cen), MRC Human Nutrition Research and the Department of Epidemiology and Public Health at the University College London Medical School. Fieldwork was carried out between February 2008 and August 2011. Details of the survey design and sampling methods of NDNS have been published elsewhere.^14^ Briefly, a random sample was drawn from the Postcode Address File, a list of all the addresses in the UK. Addresses were clustered into primary sampling units (PSUs), small geographical areas based on postcode sectors, randomly selected from across the UK. From each PSU, 27 addresses were randomly selected, and information describing the purpose of the study was posted to the selected addresses. Interviewers then contacted these addresses to arrange a face-to-face visit to recruit participants and place diet diaries (see online supplementary figure S1). To ensure that seasonal effects do not confound the sample profile, the sample of postcode sectors were systemically allocated on a monthly basis so that each quarter's allocation gives an unbiased sample of the UK. Overall, 3073 survey members completed three or four dietary recording days, giving a response rate of 55% in year 1, 55% in year 2 and 52% in year 3, respectively. The summary of achieved response rates at the household level and reasons for unproductive responses have been described in detail for the NDNS rolling programme years 1 to 3 in the published report.^15^

This survey was conducted according to the guidelines set in the Declaration of Helsinki. All procedures involving human volunteers were approved by the Oxfordshire Research Ethics Committee and all participants gave informed consent.

### Biomarkers of glucose status

All individuals who completed 3 or 4 days of dietary recording were eligible for a nurse visit. Of these, 74% adults and 42% children provided a blood sample.^16^ The differences in sociodemographic factors between those who did or did not provide blood samples have been described in detail in Appendix B of the NDNS rolling programme report published by the Department of Health.^15^ Briefly, a non-response analysis was conducted separately for children and adults. Adults from a non-White ethnic background, who were home owners or buying with a mortgage, who did not work, were younger (19–30 years) and male and those with poor general health (self-reported) were less likely to give a blood sample. The analysis for children showed a strong age difference; young children were far less likely to give blood, with males under the age of four years being the least likely. Children whose parents did not work, who were from a non-White ethnic background and who lived in owner-occupied accommodation were also less likely to give blood. Conversely, children with poor general health were more likely to give a blood sample. The above characteristics were used to generate the blood sample non-response weights. Region, household size and some other response behaviours were also included in the model as control variables. The weights therefore adjust the responding sample to make it more representative of all eligible individuals. Fasting blood samples were collected for measurements of Hb_A1c_ and glucose. The volume of blood collected varied by age, with 33 mL being taken from adults and children aged ≥16 years, 19.5 mL from those aged 7–15 years and 10 mL for children aged 1.5–6 years. Children aged ≤3 years were not required to fast. For Hb_A1c_, blood was drawn into EDTA-tubes. The samples were posted by the nurse on the day of collection and Hb_A1c_ was analysed within 24 h of sampling in the routine UK National Health Service Laboratory at Addenbrooke's Hospital, Cambridge, UK. Optimal concentrations of Hb_A1c_ were defined as Hb_A1c_ <6.5%, while high (or diabetic) Hb_A1c_ was defined by values ≥6.5%.

For glucose, blood was collected into fluoride tubes. Because insufficient blood (10mL) was taken in the youngest children aged 1.5–3 years, glucose was not analysed in this age group. Tubes were taken by the nurse on the day of collection to one of the field laboratories, where they were immediately processed then stored below−40°C before being transported in batches on dry ice to the Human Nutrition Research, Cambridge, UK, for analysis. Glucose samples were then analysed using a Dade Behring Dimension analyser (Dade Behring, Deerfield, Illinois, USA). Consistency of measurements was checked by running three controls on each run. Fasting glucose concentrations <6.1 mmol/L were considered normal. Survey members with fasting glucose between 6.1 and 6.9 mmol/L were deemed to have impaired fasting glucose. High (or diabetic) glucose concentrations were defined by concentrations ≥7 mmol/L. Hb_A1c_ and glucose cut-offs were selected as the WHO recommendations for diagnosis of diabetes. Self-reported diabetes was collected as part of the nurse visit using the Computer-Assisted Personal Interviewing (CAPI) questionnaire that asked survey members whether they had any long-standing illness or disability. Survey members who reported having diabetes including hyperglycaemia were assigned a code which was then used to identify survey members with self-reported diabetes. All nurses received training and information on the fieldwork quality control had been published previously.^15^

### Statistical analyses

Results are presented by sex and age groups (1–3 years, 4–10 years, 11–18 years, 19–64 years and ≥65 years). Data were weighted to correct for differential non-response in giving a blood sample, since individuals who refused or were unable to provide a blood sample could differ from individuals who provided blood samples. The weighting factor matched participants to the general population distribution in terms of age, sex and Government Office Region. It also matched the weighted participants to the individual questionnaire in terms of household size, ethnicity of the main food provider and economic activity of the household reference person. Details of the statistical methods used to generate the weighting factor have been described in detail elsewhere.^17^ χ^2^ Tests were used to determine the difference in Hb_A1c_ or glucose status according to sex and age group. All data were analysed using Predictive Analytics SoftWare V.18 (SPSS Inc, Chicago, Illinois, USA). Significance level was set at p<0.05.

## Results

General characteristics of the study sample are presented in table 1. Continuous data are presented as mean±SD and range. There were 25 cases of self-reported diabetes in adults aged ≥19 years. Of these, 25 had data for Hb_A1c_ and 22 had data for glucose, respectively. Approximately 71.4% of men and 100% of women with diabetes had Hb_A1c_ concentrations ≥6.5%, while 69.2% of men and 88.9% of women had glucose concentrations ≥7 mmol/L (tables 2 and 3).

**Table 1:** General characteristics of the study population

Variable	
Age, years	37.1±22.9 (1–91)
Weight, kg	67.1±22.2 (9.6–130)
Body mass index, kg/m^2^	24.9±5.9 (11.1–45.7)
Waist/hip ratio	0.86±0.09 (0.61–1.15)
Glycosylated haemoglobin (Hb_A1c_, %)	5.51±0.58 (3.7–11.7)
Glucose, mmol/L	5.12±1.15 (2.54–21.24)
Self-reported diabetes or hyperglycaemia, n (%)	26 (8.0)
Sex, n (%)	
Male	516 (46.3)
Female	599 (53.7)
Age group, years, n (%)	
1.5–3	21 (1.9)
4–10	117 (10.5)
11–18	250 (22.4)
19–64	575 (51.6)
65+	152 (13.6)
Region, n (%)	
England: North	251 (22.5)
England: Central/Midlands	212 (19.0)
England: South (including London)	455 (40.8)
Scotland	95 (8.5)
Wales	60 (5.4)
Northern Ireland	42 (3.8)
Ethnic group, n (%)	
White	1024 (91.8)
Mixed ethnic group	17 (1.5)
Black or Black British	23 (2.1)
Asian or Asian British	37 (3.3)
Any other group	14 (1.3)
National Statistics Socioeconomic Classification, n (%)	
Higher managerial and professional occupations	169 (15.2)
Lower managerial and professional occupations	317 (28.5)
Intermediate occupations	103 (9.3)
Small employers and own account workers	111 (10.0)
Lower supervisory and technical occupations	109 (9.8)
Semi-routine occupations	143 (12.9)
Routine occupations	115 (10.3)
Never worked	26 (2.3)
Other	19 (1.7)
Smoking history, n (%)	
Current cigarette smoker	180 (21.5)
Ex-regular cigarette smoker	176 (21.1)
Never regular cigarette smoker	480 (57.4)

Continuous variables are presented as means±SD (range).

**Table 2:** Glycosylated haemoglobin (Hb_A1c_) in survey members with self-reported diabetes in the National Diet and Nutrition Survey (NDNS) rolling programme—Y1–Y3 (n=25)

	Unweighted, n	Mean	95% CI	<6.5%	≥=6.5%
Male (years)					
19–64	6	6.8	(6.1 to 7.5)	3 (50%)	3 (50%)
65+	8	7.3	(6.9 to 7.7)	1 (12.5%)	7 (87.5%)
Female (years)					
19–64	7	8.0	(6.7 to 9.3)	0 (0%)	7 (100%)
65+	4	7.7	(6.9 to 8.5)	0 (0%)	4 (100%)

Unweighted data.

**Table 3:** Fasting glucose in survey members with self-reported diabetes in the National Diet and Nutrition Survey (NDNS) rolling programme—Y1–Y3 (n=22).

	Unweighted, n	Mean	95% CI	<6.1 mmol/L	≥=6.1 and <7 mmol/L	≥=7 mmol
Male, years						
19–64	4	8.0	(4.5 to 11.5)	2 (50%)	0 (0%)	2 (50%)
65+	9	8.6	(7.2 to 10)	1 (11.1%)	1 (11.1%)	7 (77.8%)
Female, years						
19–64	5	12.1	(6.9 to 17.3)	0 (0%)	1 (20%)	4 (80%)
65+	4	10.2	(7.5 to 12.9)	0 (0%)	0 (0%)	4 (100%)

Unweighted data.

After excluding survey members with self-reported diabetes, there were 1016 results for Hb_A1c_ and 942 for glucose (not the same individuals in each case). A summary of Hb_A1c_ and glucose concentrations in the combined Y1–Y3 NDNS rolling programme is provided in tables 4 and 5.

**Table 4:** Glycosylated haemoglobin (Hb_A1c_) in the National Diet and Nutrition Survey (NDNS) rolling programme—Y1–Y3 (n=869)

	Weighted, n	Unweighted, n	Mean	95% CI	<6.5%	≥=6.5%
Male, years						
1.5–3	15	11	5.4	(5.3 to 5.5)	15 (100%)	0 (0%)
4–10	63	61	5.3	(5.2 to 5.4)	63 (100%)	0 (0%)
11–18	101	128	5.3	(5.2 to 5.4)	101 (100%)	0 (0%)
19–64	270	222	5.5	(5.4 to 5.6)	266 (98.3%)	4 (1.7%)
65+	50	49	6.1	(5.8 to 6.4)	42 (83.6%)	8 (16.4%)
Female, years						
1.5–3	13	10	5.6	(4.9 to 6.3)	12 (88.7%)	2 (11.3%)
4–10	59	47	5.3	(5.2 to 5.4)	59 (100%)	0 (0%)
11–18	96	107	5.3	(5.2 to 5.4)	96 (100%)	0 (0%)
19–64	269	305	5.4	(5.4 to 5.4)	262 (97.6%)	6 (2.4%)
65+	74	76	5.8	(5.7 to 5.9)	73 (98.5%)	1 (1.5%)

**Table 5:** Fasting glucose in the National Diet and Nutrition Survey (NDNS) rolling programme—Y1–Y3 (n=869)

	Weighted, n	Unweighted, n	Mean	95% CI	<6.1 mmol/L	≥=6.1 and <7 mmol/L	≥=7 mmol
Male, years							
4–10	42	45	4.8	(4.7 to 4.9)	42 (100%)	0 (0%)	0 (0%)
11–18	99	127	4.8	(4.7 to 4.9)	99 (100%)	0 (0%)	0 (0%)
19–64	252	209	5.2	(5.1 to 5.3)	230 (91.2%)	14 (5.4%)	9 (3.4%)
65+	51	49	6.2	(5.7 to 6.7)	38 (74.4%)	3 (5.1%)	10 (20.5%)
Female, years							
4–10	37	34	4.7	(4.6 to 4.8)	37 (100%)	0 (0%)	0 (0%)
11–18	91	103	4.7	(4.6 to 4.8)	91 (100%)	0 (0%)	0 (0%)
19–64	256	298	5.0	(4.9 to 5.1)	246 (96%)	4 (1.7%)	6 (2.3%)
65+	73	77	5.1	(4.9 to 5.3)	67 (92%)	4 (5.9%)	2 (2.1%)

The distribution of Hb_A1c_ and glucose in NDNS is also presented in table 6. The results for glucose show that for adults aged 19–64 years, roughly 5.4% of the men and 1.7% of the women sampled had impaired fasting glucose; the figures for men aged 65 years and over were similar to the 19–64 years age group with nearly 5.1% of men having glucose concentrations between 6.1 and 6.9 mmol/L. Only 5.9% of women ≥65 years had glucose concentrations between 6.1 and 6.9 mmol/L. Over 20% of men aged ≥65 years had fasting glucose concentrations above the clinical cut-off for diabetes (≥7 mmol/L) in comparison to 2.1% of women of similar age (P for χ^2^=0.007). The figures were much lower for men aged 19–64 years (3.4% of men) compared with the ≥65 years age group. Around 2.3% of women aged 19–64 years had fasting glucose concentrations ≥7 mmol/L. Hb_A1c_ concentrations followed a similar trend to glucose concentration in the 65 years and over age group, with 16.4% of men having Hb_A1c_ concentrations ≥6.5%, compared to 1.5% of women (P for χ^2^=0.003). Children and teenagers had fasting glucose and Hb_A1c_ values largely within the normal range.

**Table 6:** Distribution of glycosylated haemoglobin (Hb_A1c_) and glucose in the National Diet and Nutrition Survey (NDNS) rolling programme—Y1–Y3

	Centile			
	5	25	75	95
*Hb_A1c_*				
Male, years				
1.5–3	5	5.2	5.6	5.7
4–10	4.9	5.2	5.5	5.7
11–18	4.8	5.1	5.5	5.6
19–64	4.9	5.2	5.7	6.3
65+	5.3	5.6	6	7.9
Female, years				
1.5–3	4.7	5	5.4	8.9
4–10	4.8	5.1	5.4	5.6
11–18	4.7	5.1	5.6	5.8
19–64	4.8	5.2	5.7	6.1
65+	5.1	5.6	6.1	6.4
*Glucose*				
Male, years				
4–10	4.33	4.59	4.91	5.24
11–18	4.34	4.59	4.99	5.47
19–64	4.25	4.76	5.4	6.46
65+	4.38	5.03	6.32	10.41
Female, years				
4–10	4.26	4.5	4.86	5.11
11–18	4.15	4.51	4.92	5.23
19–64	4.22	4.62	5.2	5.84
65+	4.29	4.69	5.39	6.3

## Discussion

The present study reports Hb_A1c_ and glucose results for the NDNS rolling programme (2008–2011). The findings show that there are sex-related and age-related differences in Hb_A1c_ and glucose distribution. Overall, the prevalence of Hb_A1c_ concentrations ≥6.5% was higher in men aged ≥65 years compared to the rest of the population. Similarly, 20.5% of men aged ≥65 years had fasting glucose concentrations above the clinical cut-off for diabetes (≥7 mmol/L). Together these findings indicate a high prevalence of undiagnosed diabetes in elderly men. These figures are greater than the 5% prevalence estimates reported by the Quality and Outcome Framework Achievement Data in recent successive years. What is of concern, however, is the large proportion of men aged 19–64 years (5.4%) who have fasting glucose concentrations between 6.1 and 6.9 mmol/L. The fasting glucose concentrations within this range are indicative of impaired glucose tolerance, a risk factor for the development of type 2 diabetes. This implies that the prevalence of undiagnosed diabetes could potentially increase in the UK.

Our findings also indicate that the proportion of individuals with self-reported diabetes who have Hb_A1c_ or glucose concentrations above the optimal cut-offs is high. This finding is in agreement with data from HSE which showed that, despite considerable improvement in the management of diabetes in England, less than half of the individuals with diagnosed diabetes achieve treatment targets for glycaemic control (Hb_A1c_ ≤7%).^13^ Elevated Hb_A1c_ and glucose concentrations are indications of poor glycaemic control, which can be associated with numerous diabetes-related complications including macrovascular and microvascular complications such as retinopathy and nephropathy.^10^ ^11^ Recent studies have also shown that elevated Hb_A1c_ is related to new onset CVD even in individuals without diabetes.^18^ In fact, according to findings from the North West Adelaide Health Study, the OR for new onset CVD is 2.5% higher in individuals with Hb_A1c_>5.4% compared to those with Hb_A1c_≤5%.^18^ Poor glycaemic control is known to be a major predictor of CVD morbidity and mortality in individuals with type 2 diabetes. When combined with other CVD risk factors and diabetes-related risk factors, the risk for CVD mortality increases further.^19^ Taken together, the findings from the current NDNS rolling programme can carry important public health implications and indicate the need to improve monitoring of Hb_A1c_ and glucose in the UK population. The underlying personal and social-environmental barriers to diabetes management in men and the elderly will also need to be identified.

In the present analyses of NDNS, 3.4% of men and 2.3% of women aged 19–64 years were found to have glucose concentration above 6.9 mmol/L. The proportion of men with undiagnosed diabetes increased with age to over 20% but not in women (2.1%). The above results are comparable to the findings from NHANES 1999–2002 wherein 26%, 2.8% and 6.5% of adults aged >20 years had impaired fasting glucose (>5.6 mmol/L), undiagnosed diabetes (fasting glucose >7 mmol/L) and diagnosed diabetes, respectively.^20^ However, in NHANES, the prevalence of undiagnosed diabetes in elderly men was lower (7.9%) compared to our study.^20^ We did not investigate the factors that may have contributed to sex and age differences in the prevalence of undiagnosed diabetes, particularly differences in body composition and diet, as it was beyond the purpose of the current study. Moreover, the limited number of diabetes cases in the present study renders it difficult to conduct more detailed multivariate analysis. Ageing and disturbances in glucose regulation share similar cellular pathways that are linked to obesity.^21^ Likewise, oestrogen and its receptors play a key role in the regulation of insulin sensitivity, which may explain some of the observed sex differences.^22^

We acknowledge the relatively small sample size as a limitation of the study. Hb_A1c_ concentration was measured in 352, 350 and 339 survey members in years 1, 2 and 3, respectively. For glucose, there were 324 measures in year 1, 338 measures in year 2 and 302 measures in year 3. Nevertheless, data have been weighted in the present analysis taking into account differences in sociodemographic characteristics between the survey sample and the total population in the UK in terms of age by sex and Government Office Region. This suggests that results from the present analysis should be representative of the UK population as a whole. Moreover, unlike previous surveys, NDNS includes data from individuals from areas of high deprivation, which are generally less likely to have Hb_A1c_ measured. NDNS is also one of the few studies where it will be possible to monitor temporal trends in Hb_A1c_ and glucose concentrations in the UK. This is because NDNS is planned to run on a rolling basis. In future, given a larger sample size, it will be possible to study the major determinants of diabetes risk in the UK population. Although the implications of the current study remain to be elucidated, inclusion of Hb_A1c_ and glucose measurements in the NDNS rolling programme is likely to form the basis for examining the role of diet and changes in diet over the life course of diabetes risk. The differences in diet and dietary patterns between men and women will also need to be investigated and the implication of varying dietary trajectories on age-related changes in Hb_A1c_ and glucose will subsequently need to be clarified in longitudinal studies.

In conclusion, the present study provides preliminary data on the distribution of Hb_A1c_ and glucose concentrations in a nationally representative UK population. With the prevalence of type 2 diabetes projecting a rise, NDNS will provide a unique opportunity for monitoring temporal trends and assessing the associations between diet and diabetes risk in the UK.

**What is already known on this subject**

- In England, the number of people diagnosed with diabetes has risen. Several surveys provide information on glucose status in the UK. However, most surveys are limited to one UK country and to one age group.

**What this study adds**

- This study provides descriptive data on the distribution of biomarkers of glucose status in a representative UK sample covering all UK countries and all ages from 1.5 years and upwards.

## Supplementary Material

Web figure
