# Supplementary figures and images for: Biomarkers of diabetes risk in the National Diet and Nutrition Survey rolling programme (2008–2011)
Source: J Epidemiol Community Health. 2013 Sep 19;68(1):51–6. doi: 10.1136/jech-2013-202885 (PMC3888635; doi:10.1136/jech-2013-202885)

**Supplementary Figure 1.** Diagram showing the structure and design of the NDNS rolling programme.

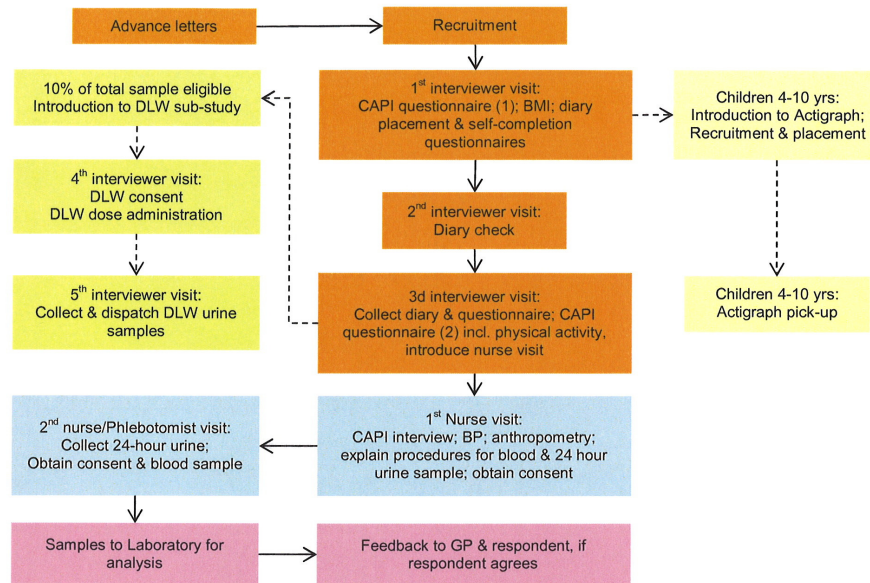

Supplement: Web figure [file jech-2013-202885-s1.pdf]
